# Complications and Treatment Challenges After Metallic Airway Stenting in a 10‐Year‐Old Child With a Malignant Mediastinal Tumour

**DOI:** 10.1002/rcr2.70411

**Published:** 2025-11-23

**Authors:** Ryosuke Higuchi, Fumihiko Kinoshita, Tetsuzo Tagawa, Utako Oba, Yuhki Koga, Atsushi Wakizono, Taichi Matsubara, Mikihiro Kohno, Keigo Ozono, Tomoyoshi Takenaka, Tomoharu Yoshizumi

**Affiliations:** ^1^ Department of Surgery and Science, Graduate School of Medical Sciences Kyushu University Fukuoka Japan; ^2^ Department of Thoracic Surgery Kyushu University Hospital Fukuoka Japan; ^3^ Department of Thoracic Surgery, Clinical Research Institute, National Hospital Organization Kyushu Medical Center Fukuoka Japan; ^4^ Department of Pediatrics, Graduate School of Medical Sciences Kyushu University Fukuoka Japan; ^5^ Department of Pediatrics National Hospital Organization Kyushu Cancer Center Fukuoka Japan

**Keywords:** granulation, metallic stent, retention suture

## Abstract

A 10‐year‐old patient underwent metallic stent implantation in the trachea and right main bronchus for tracheal stenosis caused by extramural compression from a posterior mediastinal malignant rhabdoid tumour. Following chemoradiotherapy, the tumour entered remission. One year and 6 months after stent placement, refractory granulation tissue formed in the tracheal stent. The stent was removed using a rigid bronchoscope under general anaesthesia with extracorporeal membrane oxygenation to prevent granulation recurrence. Thereafter, no recurrence of granulation occurred in the tracheal stent within 2 years; however, refractory granulation developed in the right main bronchial stent. Removal of the right main bronchial stent was considered. However, as sputum frequently adhered to the retention suture of the stent, the retention suture was removed using a flexible bronchoscope. Since then, there has been no granulation for more than 10 months.

## Graphical abstract

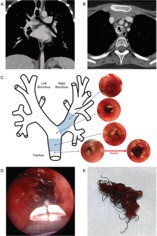

## 1 Introduction

We report a case of refractory granulation occurring 7 years after metallic stent implantation in the trachea and right main bronchus of a 10‐year old patient with tracheobronchial stenosis caused by a malignant posterior mediastinal tumour. The tracheal stent was ultimately removed with great effort due to refractory granulation tissue. However, subsequent granulation in the right main bronchial stent was managed by removing the stent's retention suture—a safer and less invasive approach that prevented recurrence.

## 2 Case Report

**FIGURE 1:**
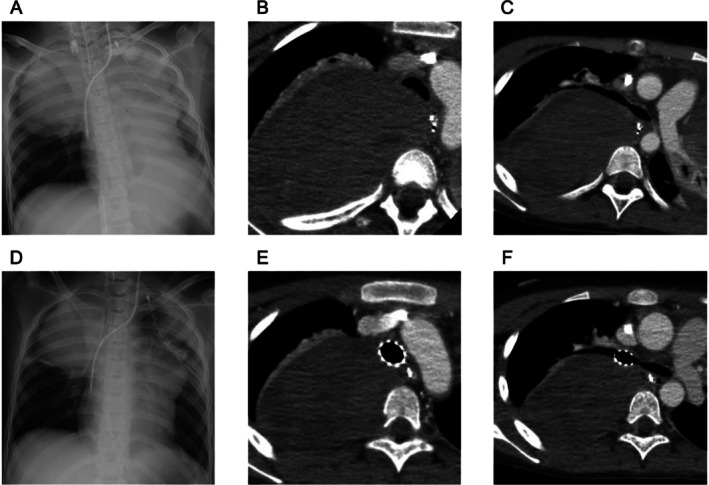
Chest radiograph reveals a large right upper lung zone mass measuring 83 mm × 75 mm with extensive bilateral atelectasis (A). Computed tomography (CT) imaging demonstrates compression and stenosis of the trachea and right main bronchus by the tumour (B, C). Two metallic stents were implanted in the trachea and right main bronchus (D–F).

A 10‐year‐old girl was referred to our hospital with cough and respiratory distress. Chest radiography revealed a large posterior mediastinal mass (Figure 1A) and computed tomography (CT) demonstrated compression of the trachea and right main bronchus with extensive bilateral atelectasis (Figure 1B,C). Because of unstable respiratory status, the patient was intubated with a double‐lumen tube, and placed on extracorporeal membrane oxygenation (ECMO). Histopathology confirmed malignant rhabdoid tumour.

Because the expected prognosis was only a few weeks, two metallic stents were implanted to facilitate ECMO withdrawal and extubation, allowing the patient to spend her final days at home (Figure 1D–F). We used Ultraflex uncovered stents (Boston Scientific, USA): 14 × 30 mm for the trachea and 12 × 30 mm for the right main bronchus. Stent sizes were determined by CT and the stents were placed using a flexible bronchoscope under fluoroscopic guidance. ECMO was gradually discontinued, and the patient was extubated approximately 2 weeks later.

Fortunately, the patient responded to chemoradiotherapy, and the tumour entered remission after 10 months. Given the risk of tumour recurrence and the hazards of stent removal, the stents were left in place.

**FIGURE 2:**
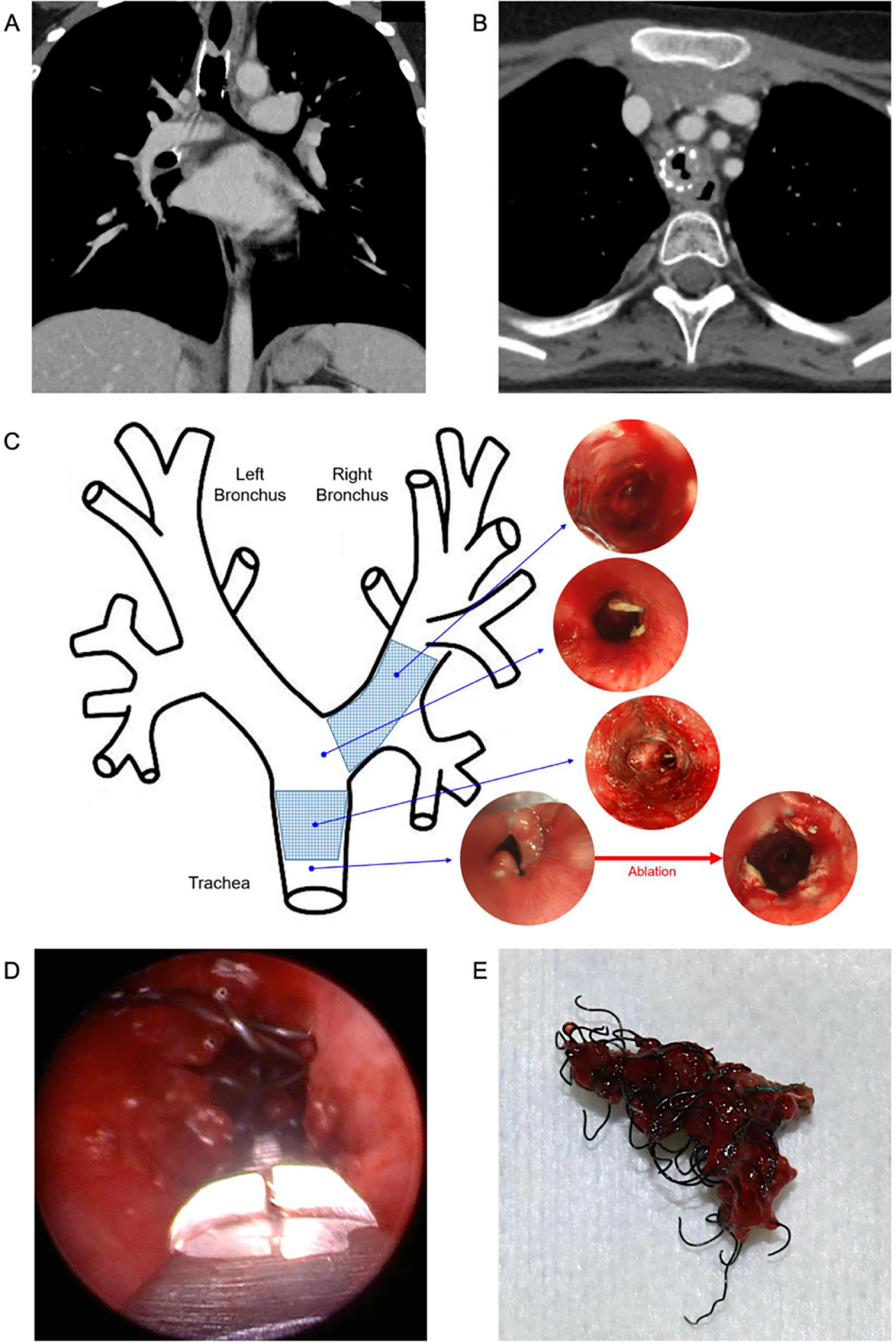
Granulation tissue in the tracheal stent (A, B). Bronchoscopic microwave ablation and bronchial balloon dilation were performed (C). The tracheal stent was removed under general anaesthesia using rigid bronchoscopy (D, E).

Granulation developed in the tracheal stent 1 year and 6 months after stent placement (Figure 2A,B). Despite inhaled steroids, ablation and balloon dilation (Figure 2C), symptoms relapsed within 1 month. Refractory granulation required bronchoscopic treatment every 2 months, for a total of seven sessions. At the third ablation, stent removal was deemed necessary, and preparations for surgery were made. The patient underwent tracheal stent removal under general anaesthesia and ECMO using a rigid bronchoscope. The metallic stent, firmly embedded in the tracheal wall, was removed piecemeal using forceps (Figure 2D,E). The procedure lasted 8 h and 31 min. Because the right main bronchial stent had not induced granulation tissue and the patient was asymptomatic, it was left in place. The patient was extubated on postoperative day 7 and discharged on day 11.

**FIGURE 3:**
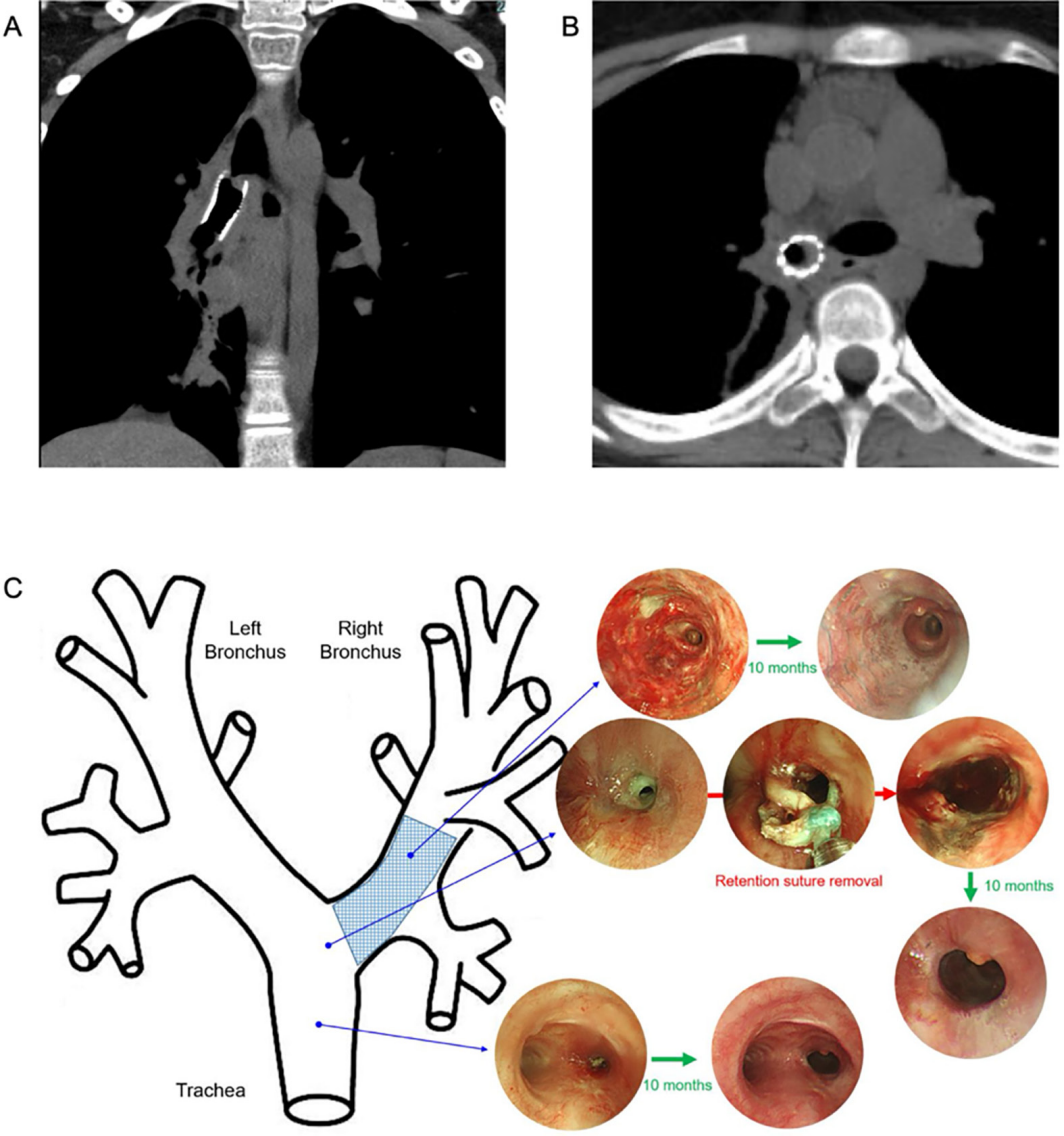
Granulation tissue in the right main bronchial stent (A, B). The retention suture at the entrance of the right main bronchial stent was removed using flexible bronchoscopy. No recurrence of granulation was observed 10 months after suture removal (C).

Subsequently, the patient's condition improved, with no granulation recurrence for 2 years. However, granulation tissue developed in the right main bronchial stent (Figure 3A,B). Bronchoscopic microwave ablation, balloon dilation, inhaled steroids and topical application of mitomycin C provided only temporary relief; the granulation was refractory and bronchoscopic treatment was required every 1–2 months. Removal of the right main bronchial stent was considered; however, risks were expected to be comparable to those of tracheal stent removal. Because frequent sputum adherence and granulation formation were observed near the retention suture at the entrance of the right main bronchial stent, the suture was removed using a flexible bronchoscope. The hardened suture could not be cut with scissors or ablated with an argon plasma coagulator; therefore, it was gradually torn off with biopsy forceps (Figure 3C). Ten months after suture removal, bronchoscopy revealed no granulation (Figure 3C) and the patient remains under observation without further treatment.

## 3 Discussion

Central airway stenosis due to benign or malignant diseases causes severe dyspnea and significantly reduces the patient's quality of life. Although surgical treatments are available, airway stenting remains an important therapeutic strategy to improve respiratory function [1, 2]. Metallic stents can be easily implanted using a soft bronchoscope under fluoroscopic guidance, but they are frequently associated with tumour ingrowth and granulation tissue formation, reported in 15%–20% of cases [3]. Although argon plasma coagulation and cryotherapy are also effective treatments, they were omitted in this case because microwave ablation provided sufficient temporary improvement.

In addition, metallic stents become significantly more difficult to remove following long‐term implantation, and many complications have been reported [4]. Malignant rhabdoid tumour carries an extremely poor prognosis [5], and in this case the patient's prognosis was expected to be only a few days. Moreover, metallic stents were readily available, while preparation of silicone stents required several weeks. Therefore, we selected a metallic airway stent to enable rapid weaning from ECMO and ventilator. Surgical stent removal was also considered; however, extensive thoracic adhesions caused by the tumour made this approach unfeasible. Consequently, the tracheal stent was removed using a rigid bronchoscope.

The Ultraflex metallic stent consists of woven Nitinol with end loops secured by a retention suture. In our patient, granulation consistently developed at the site of sputum adherence to the suture. Granulation tissue is believed to arise from both mechanical irritation and inflammation caused by bacterial infection [6]. Removing the suture eliminated sputum retention and halted granulation recurrence. While the precise mechanism remains unclear, this case suggests that retention sutures may play a critical role in refractory granulation.

In conclusion, this case demonstrates that removal of a stent's retention suture can be an effective, minimally invasive treatment for managing refractory granulation after metallic stent implantation. Metallic stents in children must be considered strictly as a last‐resort, short‐term solution or used only when alternatives are unavailable. Early planning for removal or replacement is essential to avoid long‐term complications in paediatric patients with developing airways.

## Author Contributions

R.H. drafted the manuscript. F.K. and T.T. critically revised the manuscript. All authors read and approved the final manuscript.

## Consent

The authors declare that written informed consent was obtained for the publication of this manuscript and accompanying images using the consent form provided by the Journal.

## Conflicts of Interest

The authors declare no conflicts of interest.

## Data Availability

Research data are not shared.
